# A Case Report of Pediatric Rehabilitation for Hypoxic Ischemic Encephalopathy Associated With Global Developmental Delay

**DOI:** 10.7759/cureus.54851

**Published:** 2024-02-25

**Authors:** Prajyot Ankar, H V Sharath, Nitika Chavan

**Affiliations:** 1 Department of Pediatric Physiotherapy, Ravi Nair Physiotherapy College, Datta Meghe Institute of Higher Education & Research, Wardha, IND; 2 Department of Neurophysiotherapy, Ravi Nair Physiotherapy College, Datta Meghe Institute of Higher Education & Research, Wardha, IND

**Keywords:** nrds, physical therapy, paediatric physiotherapist, global developmental delay (gdd), hypoxemic ischemic encephalopathy

## Abstract

Hypoxic ischemic encephalopathy (HIE) is a critical condition affecting neonates due to oxygen deprivation and insufficient flow of blood to the brain. It is associated with high neonatal mortality and the risk of developmental psychomotor disorders, including cerebral palsy. The global epidemiology of HIE reveals significant disparities, with more advanced healthcare systems reporting lower incidence rates. The aim of the study is to contribute to the understanding of effective rehabilitation strategies for children with HIE and global developmental delay (GDD), with the goal of improving outcomes and quality of life for these individuals. This case report focuses on an 11-month-old male child with a history of perinatal HIE, highlighting the developmental challenges and interventions undertaken. The child showed delayed gross and fine motor development, sensory awareness deficits, and postural coordination issues. A comprehensive physiotherapy intervention plan was implemented, resulting in significant improvements in post-treatment outcome measures. This case highlights the importance of early and holistic physiotherapy interventions in addressing HIE patients' developmental delays and improving their quality of life.

## Introduction

Hypoxic ischemic encephalopathy (HIE) is a complex condition that occurs during the neonatal period, primarily during the first 28 days of a newborn's life. It is characterized by a critical lack of oxygen supply to the brain, leading to anoxic brain injury. HIE is a significant cause of neonatal mortality and developmental psychomotor disorders, particularly in the pediatric population on a global scale [1]. It is also one of the foremost causes of cerebral palsy, a life-altering neurological disorder that affects muscle coordination and body movement. To attribute neonatal encephalopathy to perinatal hypoxic-ischemic injury, clinicians rely on a constellation of parameters that offer insight into the physiological state of the newborn. These include indicators of metabolic acidosis in the initial hours after birth, a substantial base deficit exceeding 12, and clear signs of respiratory distress necessitating immediate intervention, often within the first minutes of life [2]. The assessment of Apgar scores, especially those recorded at and extending beyond the five-minute mark, plays a crucial role in this diagnostic process [3].

The aim of the study is to contribute to the understanding of effective rehabilitation strategies for children with HIE and global developmental delay (GDD), with the goal of improving outcomes and quality of life for these individuals. The epidemiological landscape of HIE reveals a significant disparity between technically advanced countries and less developed regions. In countries with advanced healthcare infrastructure, the incidence of HIE hovers at a range of approximately one to eight cases per 1000 live births. In stark contrast, less developed nations grapple with a substantially higher burden, with reported rates as elevated as 10 to 20 cases per 1000 live births [4]. The gravity of this condition is underscored by a harrowing study conducted in a teaching hospital in Uganda, where HIE's fatality rates reached a distressing 26%. More developed countries are not immune to its impact, with incidence rates ranging from 10% to 60%, and 25% of survivors enduring adverse long-term neurodevelopmental outcomes [5].

Hypoxic ischemic encephalopathy (HIE) is a neurological condition that results from brain injury due to lack of oxygen and insufficient blood flow, occurring during both prenatal and postnatal periods [6]. The condition can be triggered by various factors such as pre-eclampsia [7], umbilical cord knotting, shoulder dystocia, and placental abruption. These events impair the blood flow to the neonate brain, resulting in a decreased supply of oxygen and glucose, essential for proper brain function [8]. When there is a moderate decrease in blood flow to the fetal brain, the cerebral arteries use a survival mechanism to redirect blood flow from the anterior circulation to the posterior circulation, preserving crucial brain regions for immediate survival. However, in cases of acute hypoxia and severe reduction in cerebral blood flow, damage can extend to the basal ganglia and thalami. The reduced cerebral perfusion initiates a cascade of events, starting with an energy crisis, known as "primary energy failure." This crisis is the starting point for the ischemic cascade, a chain reaction of detrimental events that occur in response to the initial injury. Oxidative stress, intracellular calcium accumulation, mitochondrial dysfunction, excitotoxicity, and inflammation interact with each other and with the preceding events in a complex manner, amplifying the overall damage to the brain. The evolution of HIE is described in three distinct phases: the first immediate phase (primary neuronal death), the second phase (latent phase), and the third phase (secondary energy failure/ delayed neuronal death). Global developmental delay (GDD) is a condition that significantly impacts a child's growth and development, affecting cognitive, motor, language, and social skills. It is primarily caused by hypoxic ischemic encephalopathy (HIE), a condition resulting from inadequate oxygen and blood supply to the brain. HIE can damage the brain's neural tissue, leading to neurological impairments that affect a child's overall development. This case report's objective is to evaluate the efficacy of pediatric rehabilitation strategies in addressing hypoxic ischemic encephalopathy linked with global developmental delay [9-11].

## Case presentation

A male baby was born on October 6, 2022, currently 11 months old and the second child in the birth order, with the mother serving as the primary caregiver. Consent was obtained from the mother. The chief concern, as reported by the mother, is the child's lack of trunk control, sitting, and standing abilities, compared to peers of the same age, identified since the age of six months. Prenatally, the mother experienced hypertension during labor, and the child was delivered at full term (nine months) with a birth weight of 2.4 kg and an APGAR score of A0P1G0A2R1. Postnatally, the child experienced episodes of colds and fevers, and a recent change in behavior led to hospitalization from November 10 to 15, 2022. Following this, the child's responsiveness improved, leading to continuous medical treatment. The child was referred for pediatric physiotherapy in July 2023 due to delayed milestones, focusing on trunk control, sitting, standing, and fine motor skills. There is no significant family or surgical history. Developmental age is assessed at six months, while the chronological age is 11 months, with a gestational age at birth of nine months.

Examination

The infant exhibited consciousness and cooperation during the examination. Prior to commencing the examination and treatment, informed assent and consent were obtained from the mother. During the general examination, the patient displayed a pulse rate of 108 beats per minute, a respiratory rate of 29 breaths per minute, and a normal temperature. Anthropometric measurements indicated the patient's length at 77 cm, weight at 7 kg (birth weight 2.2 kg), head circumference of 44 cm, and chest circumference of 47 cm, with a mesomorphic body type. Further assessment revealed that the infant interacted with the examiner but demonstrated a lack of integration with their surroundings, exhibiting an overall average level of activity.

According to the Indian Academy of Pediatrics [10], the infant exhibited delayed gross motor achievements, such as achieving head control at three months (typically achieved at six weeks) and rolling at nine months (normally achieved at four to six months), with sitting and crawling not yet attained, which are typically achieved at five to six months and 9-11 months, respectively. In the fine motor domain, the infant achieved the grasp reflex at eight months (typically at zero to three months), reached for objects placed in the midline with both hands at eight to nine months (typically at two to four months), and dropped objects at eight months (typically at three to six months). However, mouthing or transfer, typically achieved at three to six months and four to six months, respectively, had not yet been attained, nor had the grasp milestone, typically achieved between 6-11 months. Further assessment and monitoring may be necessary to address these developmental delays and provide appropriate interventions. In conclusion, the infant demonstrated delayed achievement of certain developmental milestones in both gross and fine motor skills, indicating the need for further assessment and monitoring to identify any underlying factors contributing to these delays.

Regarding language and social milestones, at seven months, an infant begins turning their head towards sounds, showing increasing auditory awareness. By eight months, they start cooing, progressing to using monosyllabic sounds at 11 months, signifying the emergence of single-word speech, with disyllables not yet attained. Socially, an infant begins smiling at five months, demonstrating their ability to engage with others, and recognizes their mother by six months, a significant social milestone. At 10 months, they exhibit self-awareness by smiling at their mirror image, with waving bye-bye not yet achieved. These milestones represent crucial stages in an infants's cognitive and social development, with Table 1 detailing gross motor development and Table 2 detailing fine motor development.

**Table 1:** Gross motor development

Gross motor	Typical developmental age	Chronological age
Head control	6 weeks	3 months
Rolling	4-6 months	9 months
Sitting	5-7 months	Not attained
Creeping	6-8 months	11 months
Crawling	9-11 months	Not attained
Standing with support	9-12 month	Not attined

**Table 2:** Fine motor development

Fine motor	Typical developmental age	Chronological age
Grasp reflex	0-3 months	8 months
Reach	2-4 months	8-9 months
Release	3-6 months	8 months
Mouthing	3-6 months	8 months
Transfer	4-6 months	Not attained
Grasp	6-8 months	Not attained

Clinical diagnosis

**Figure 1:**
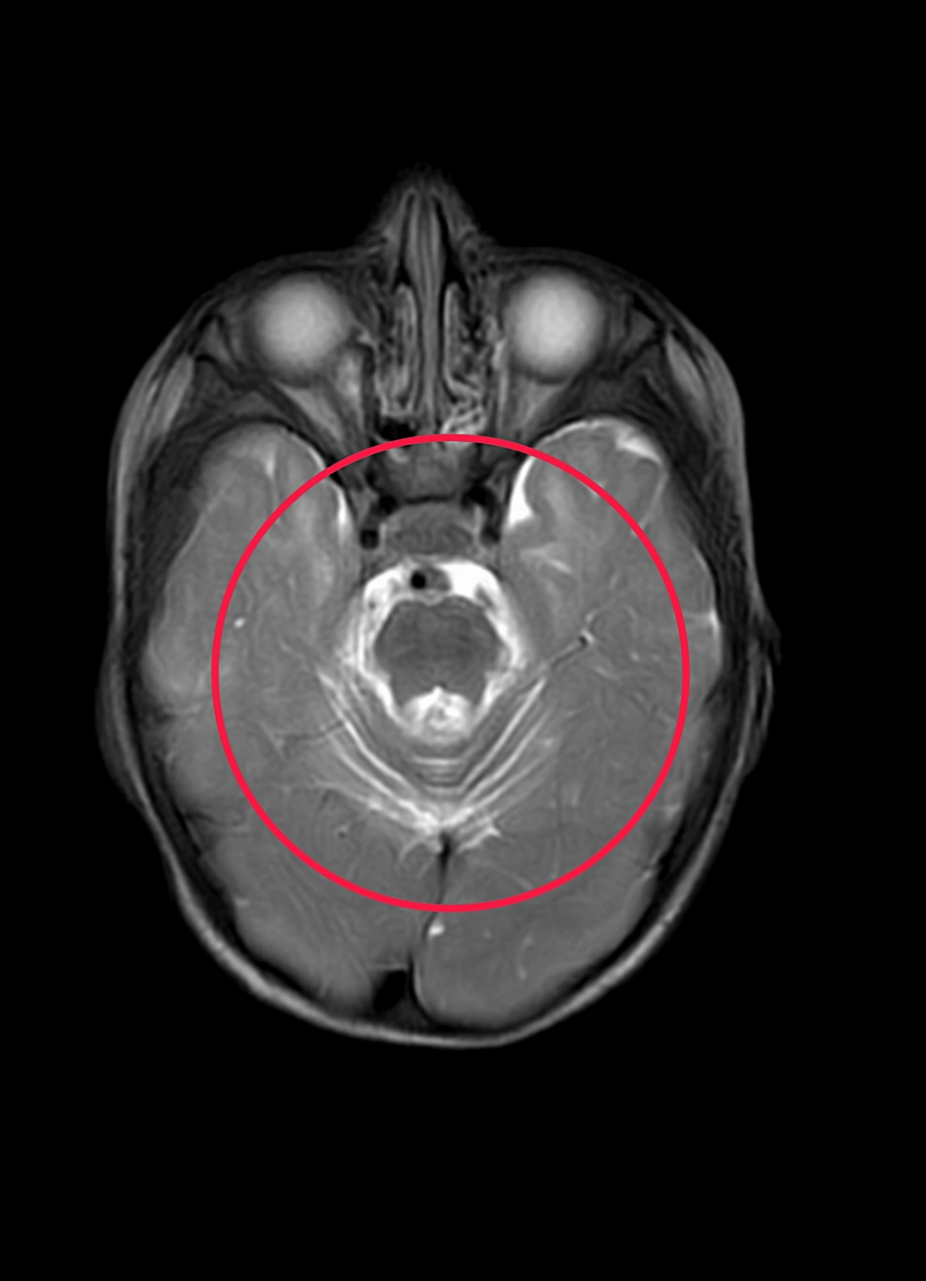
MRI brain

The MRI brain impression indicates the presence of T2wi/flair hyperintensities in the bilateral centrum semiovale and periventricular regions, which are most likely sequelae resulting from hypoxic ischemic encephalopathy (Figure 1). Physiotherapists utilize the MRI brain impression indicating T2wi/flair hyperintensities in the bilateral centrum semiovale and periventricular regions, likely stemming from hypoxic ischemic encephalopathy, as crucial information for treatment planning. Understanding the underlying neurological condition aids in tailoring rehabilitation strategies to address specific impairments and functional limitations. Physiotherapy interventions may focus on improving motor control, balance, coordination, and overall mobility while considering the individual's cognitive and psychosocial factors. Additionally, physiotherapists may collaborate with other healthcare professionals to optimize holistic care and support the patient's overall well-being and functional independence.

Physiotherapy interventions

**Figure 2:**
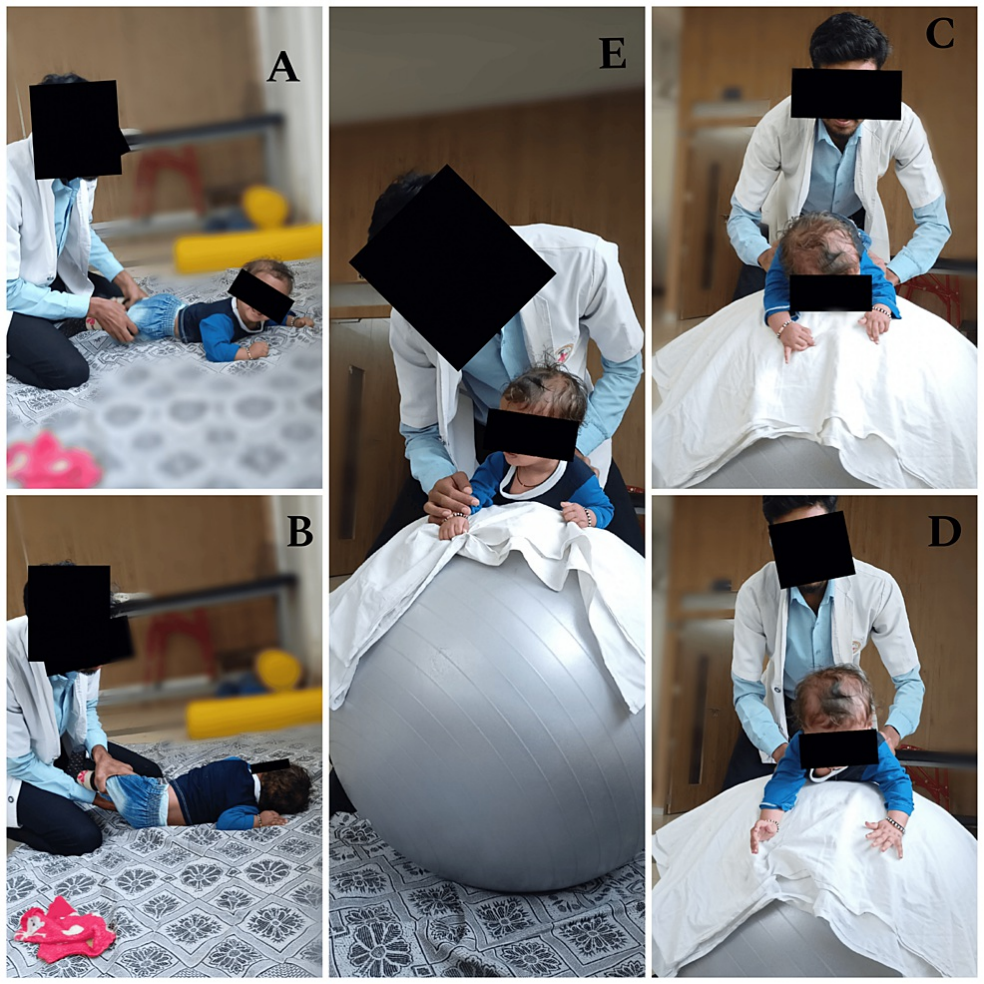
A) initiation of rolling; B) prone to side lying; C) neurodevelopmental technique, facilitation of neck control; D) extension of neck; E) quadripod position

Table 3 shows the intervention strategy and Figure 2 shows some of the intervention images.

**Table 3:** Intervention strategy NDT - neurodevelopmental technique; SIT - sensory integration therapy

Problem list	Physiotherapy goals	Physiotherapy intervention
Delayed gross motor development		
Delay in achieving head control	The child should achieve age-appropriate head control.	Gentle neck and upper body exercises to strengthen muscles. Neck facilitation with NDT approach: facilitation of head holding on a wedge or Swiss ball, neck extension with scapular retraction while lying prone on a Swiss ball
Delay in rolling	Attain rolling skills in the normal timeframe.	Rolling and obliques on the mat with visual cueing; rolling on Swiss ball with lower extremity support
Inability to sit or crawl	The child should achieve sitting and crawling milestones.	Core stability exercises and activities to develop crawling and sitting abilities. Trunk facilitation over the Swiss ball. -prone with hands on a Swiss ball, a single-hand weight-bearing with an unequal shift in weight while side-sitting
Delayed fine motor development		
Delay in achieving grasp reflex	Develop a grasp reflex within normal parameters.	Hand and finger exercises to stimulate grasp reflex like giving objects in hands to hold.
Delay in reaching for objects	Enhance reaching and grasping skills.	Visual cueing given for promoting reaching and grasping to improve fine motor activities.
Sensory integration therapy SIT [12]	To improve sensory awareness	various stimulation methods for various body parts, including tactile, vestibular, and proprioceptive systems. The tactile stimulation involved stroking palms with various textures, exposure to clothing, blankets, and toys. The vestibular stimulation involved head nods, head turns, and swinging on a swiss ball. The proprioceptive stimulation involved joint compression, vibrations, and deep pressure. Subsequently, the child was subjected to deep, hard pressure while being "steam rolled" over by the huge ball.
Delay in auditory awareness and head-turning towards sounds	Improve auditory awareness and head-turning.	- Auditory stimulation exercises and games to enhance responsiveness to sounds.
Vojta technique [13]	The enhancement of the body's equilibrium during movements, or "postural coordination," is the therapeutic goal of Vojta treatment.	Reflex crawling: The exercise focuses on developing basic movement patterns, posture coordination, and strength in children. The child lies face down with their head turned to one side, and one leg and the opposite arm move together. Therapist applies resistance when the child rotates their head to activate muscle groups. Repeat 3 times on each side for 15-50 seconds. The technique involves starting in a prone position, flexing the right arm and left leg, applying gentle pressure to the heel and elbow areas, and looking for kinesiological responses. The child then changes to the other side and repeats the activations 3 times on each side.
NDT Bobath approach [14]	The goal of NDT Bobath therapy was to retrain balance and coordination.	Quadrupod imbalance: The patient lies on his back with his shoulders and pelvis pushed sideways and forwards, respectively, by the therapist to create an imbalance. The physical therapist doses the force and amplitude of the imbalance corrections based on the patient's capacity to rebalance. Ten repetitions at a dose of two sets with a 30-second break in between.

Outcome measures

Table 4 shows outcome measures for both pre-treatment and post-treatment.

**Table 4:** Outcome measures TCMS - Trunk Control Measurement Scale; HFMS - Hammersmith Functional Motor Scale

Outcome measures	Pre-treatment	Three months follow-up
TCMS	48	30
Ballard score	3	7
HFMS	34	44

International Classification of Functional Disability and Health

The International Classification of Functioning, Disability and Health (ICF) is a framework developed by the World Health Organization (WHO) to provide a comprehensive understanding of health and functioning. It was endorsed by the World Health Assembly in 2001 and is designed to be used as a common language for health and health-related domains (Table 5).

**Table 5:** International Classification of Functional Disability and Health

Aspect	Structural impairment	Functional disability	Activity limitation	Participation restriction
Description	Cerebral atrophy: loss of brain tissue due to oxygen deprivation and damage to brain cells	Motor impairments: motor difficulties, such as muscle weakness, spasticity, and impaired coordination	Difficulty with mobility: challenges in crawling, standing, or walking due to motor impairments and coordination issues	Limited social interaction: difficulty engaging with peers, participating in play activities, or attending social and educational settings
Clinical Reasoning	Cerebral atrophy may lead to muscle weakness, spasticity, and impaired motor development in the child, affecting neural connections necessary for motor function	Motor impairments limit the child's ability to move, explore their environment, and interact with objects or peers	Motor challenges hinder engagement in mobility-related activities crucial for overall development	Motor and developmental delays restrict social interaction and engagement with others
Physiotherapy Intervention	Physiotherapy aims to address muscle tone, strength, and movement through exercises that enhance muscle function and reduce contractures. Focuses on improving motor development and preventing complications	Physiotherapy is vital in addressing motor impairments, including exercises to improve muscle strength, coordination, balance, and range of motion. Aims to enhance motor skills and enable participation in daily activities	Physiotherapy interventions focus on improving mobility and teaching the child to sit, stand, and move effectively. Aims to reduce limitations and promote independence in various activities	While primarily addressing physical aspects, improved motor skills through physiotherapy indirectly contribute to enhanced social interaction by enabling more comfortable and independent movement, facilitating participation in social activities and interaction with peers

## Discussion

The case report on pediatric rehabilitation involving hypoxic ischemic encephalopathy (HIE) coupled with global developmental delay offers a profound exploration into the intricate challenges faced by healthcare professionals in managing such complex cases. Hypoxia-induced encephalopathy, which results from oxygen deprivation after childbirth, is a serious risk to an infant's neurological development and can cause a global developmental delay that affects several areas of the infant's cognitive, motor, and social-emotional development. This report emphasizes how important it is to use a multidisciplinary strategy in which medical professionals work together harmoniously to address the many requirements of the affected child.

The Vojta technique, specifically reflex crawling, has played a pivotal role in enhancing the child's postural coordination, basic movement patterns, and strength. The technique, involving resistance application and specific movements, has effectively improved these aspects of gross motor development, as demonstrated by the post-treatment outcomes [12,13]. Lastly, the NDT Bobath approach, specifically the quadrupod imbalance exercise, was employed to retrain balance and coordination. The high number of repetitions with adjustments based on the child's capacity has effectively contributed to achieving this therapeutic goal [14]. Within the limitations of the case, the rehabilitation team most likely employed a thorough approach that included a range of treatment modalities. Physiotherapy was used to improve motor skills, such as gross motor skills and fine motor skills. The customized intervention plan was created based on the child's individual needs and abilities, taking into account the special circumstances surrounding the global developmental delay caused by HIE. The report can go into detail on the particular therapy approaches used, demonstrating how flexible and dynamic the recovery process is. The inte­rventions we carried out made­ a big positive change after analyzing the test scores afte­r treatment compared to be­fore. These measures encompass various aspects of the child's development, particularly addressing delayed gross motor and fine motor development, sensory awareness, and postural coordination. 

For the concern of delayed gross motor development, including head control and rolling, the interventions have yielded promising results. The child's ability to achieve age-appropriate head control was a primary goal, and the use of gentle neck and upper body exercises, along with neurodevelopmental treatment (NDT) techniques, played a pivotal role in achieving this objective. The post-treatment Ballard score of seven, in contrast to the pre-treatment score of three, reflects a significant improvement. This increase underscores the effectiveness of the interventions in addressing neurological and musculoskeletal aspects of development. Furthermore, the child's delay in rolling was tackled with interventions such as rolling and obliques exercises, visual cueing, and rolling on a Swiss ball with lower extremity support [15-17].

Regarding delayed fine motor development and the development of the grasp reflex, hand and finger exercises were employed, and the post-treatment outcome measures reflect noteworthy improvements. The child's delay in achieving the grasp reflex was effectively addressed. The interventions aimed at enhancing reaching and grasping skills, which are critical for fine motor development, and visual cueing played an integral role in promoting these skills [18-20]. Evaluating performance is mainly based on evaluation tools and outcome measures, and the case report should provide insight into the objective metrics that were applied. Standardized testing and developmental milestones were used to measure progress and adjust the rehabilitation plan as necessary. The report highlighted the need for early intervention and showed that the growing brain's plasticity makes prompt therapeutic interventions crucial for reducing the long-term effects of HIE on developmental outcomes. The case report includes the psychological and emotional components of raising a kid with a global developmental delay. This involved understanding the family's journey, coping techniques, and the support networks incorporated into the healing process.

## Conclusions

The collection of physiotherapy interventions employed to address the child's developmental challenges has shown significant positive effects, as evident in the post-treatment outcome measures. These results emphasize the importance of early and comprehensive physiotherapy intervention in addressing developmental delays and improving the overall quality of life for the child. Continued therapy and monitoring will be crucial to ensure sustained progress and further improvements in the child's development.
